# Water-Soluble Tomato Concentrate, a Potential Antioxidant Supplement, Can Attenuate Platelet Apoptosis and Oxidative Stress in Healthy Middle-Aged and Elderly Adults: A Randomized, Double-Blinded, Crossover Clinical Trial

**DOI:** 10.3390/nu14163374

**Published:** 2022-08-17

**Authors:** Zezhong Tian, Kongyao Li, Die Fan, Xiaoli Gao, Xilin Ma, Yimin Zhao, Dan Zhao, Ying Liang, Qiuhua Ji, Yiting Chen, Yan Yang

**Affiliations:** 1 School of Public Health (Shenzhen), Shenzhen Campus of Sun Yat-sen University, Sun Yat-sen University, Shenzhen 518107, China; 2 Guangdong Provincial Key Laboratory for Food, Nutrition and Health, Sun Yat-sen University, Guangzhou 510080, China; 3 Guangdong Engineering Technology Research Center of Nutrition Translation, Sun Yat-sen University, Guangzhou 510080, China; 4 Department of Non-Communicable Disease Prevention and Control, Shenzhen Nanshan Center for Chronic Disease Control, Shenzhen 518064, China; 5 Clinical Nutrition Department, The General Hospital of Western Theater Command, Chengdu 610000, China; 6 The Eighth Affiliated Hospital, Sun Yat-sen University, Shenzhen 518033, China

**Keywords:** water-soluble tomato concentrate, oxidative stress, platelet apoptosis, crossover clinical trial

## Abstract

Increased oxidative stress and platelet apoptotic in middle-aged and elderly adults are important risk factors for atherosclerotic cardiovascular disease (ASCVD). Therefore, it is of great significance to control the oxidative stress and platelet apoptosis in middle-aged and elderly adults. Previous acute clinical trials have shown that water-soluble tomato concentrate (WSTC) from fresh tomatoes could exert antiplatelet benefits after 3 h or 7 h, but its effects on platelet apoptosis and oxidative stress are still unknown, especially in healthy middle-aged and elderly adults. This current study aimed to examine the efficacies of WSTC on platelet apoptosis and oxidative stress in healthy middle-aged and elderly adults via a randomized double-blinded placebo-controlled crossover clinical trial (10 weeks in total). A total of 52 healthy middle-aged and elderly adults completed this trial. The results showed that WSTC could increase the serum total antioxidant capacity levels (*p* < 0.05) and decrease the serum malondialdehyde levels (*p* < 0.05) after a 4-week WSTC supplementation in healthy middle-aged and elderly adults. Platelet endogenous reactive oxygen species generation (*p* < 0.05), mitochondrial membrane potential dissipation (*p* < 0.05) and phosphatidylserine exposure (*p* < 0.05) were attenuated. In addition, our present study also found that WSTC could inhibit platelet aggregation and activation induced by collagen or ADP after intervention (*p* < 0.05), while having no effects on adverse events (*p* > 0.05). The results suggest that WSTC can inhibit oxidative stress and its related platelet apoptosis, which may provide a basis for the primary prevention of WSTC in ASCVD.

## 1. Introduction

Platelets, the most common anucleate blood cells, are essential for hemostasis, thrombosis, inflammation and atherosclerosis [1]. Animal experiments indicated that platelets are major contributors of vessel occlusion critical for cardiovascular events [2]. Accumulating evidence has shown that platelet apoptosis facilitated the progression of atherosclerotic cardiovascular disease (ASCVD) [3,4]. In different pathophysiological conditions (such as aging or dyslipidemia), the level of oxidative stress increases, thus further inducing the excessive apoptosis of platelets [5,6]. Oxidative stress and excessive apoptosis of platelets can exacerbate the risk of ASCVD [7,8,9].

A previous cross-sectional study [6] has shown that there was increased oxidative stress and platelet apoptotic markers in middle-aged and elderly adults, and further mice studies implicated a change in oxidative stress as the mechanism [6]. Mechanistic studies revealed that excessive reactive oxygen species (ROS) stimulated the expression and activation of pro-apoptosis proteins in the Bcl-2 family and promoted the translocation of pro-apoptosis proteins to the mitochondria [10,11,12]. Then, depolarization of the mitochondrial membrane potential (ΔΨm) is initiated to form the mitochondrial permeability transition pore (mPTP), which leads to phosphatidylserine (PS) exposure and, ultimately, platelet apoptosis [10,11]. Excessive abnormal platelet apoptosis may increase the formation of PS-positive platelets and microparticles (MPs), which leads to increased procoagulant activity and thrombosis enhancement [13,14,15]. In addition, a cross-sectional study found that ROS production of platelets was strictly correlated with high platelet reactivity [16]. Therefore, controlling platelet apoptosis and oxidative stress can be applied for the early prevention of ASCVD in middle-aged and elderly adults.

A previous study has shown that a suitable diet, such as the Mediterranean diet, could attenuate platelet-related mortality in older adults at high cardiovascular risk [17]. Epidemiological studies suggested that tomato and tomato product consumption were associated with a reduced risk for ASCVD, which might be partly due to tomatoes containing substances with antiplatelet properties [18,19,20]. A large prospective study showed that there was little evidence for an overall association between dietary lycopene and the risk of ASCVD [21]. Another epidemiological study found there was no beneficial effect of higher plasma lycopene levels on myocardial infarction [22]. These indicated that other water-soluble ingredients in tomatoes might play an important role in decreasing the risk of ASCVDs. Water-soluble tomato concentrate (WSTC) is a concentrated tomato product via removing fat-soluble ingredients in tomatoes (Lycopersicon esculentum), which is primarily comprised of nucleoside derivatives, phenolic conjugates, flavonoid derivatives, and quercetin derivatives [23]. Previous studies in vitro have shown that WSTC exerted potent regulatory effects on the platelet function, such as platelet activation and aggregation inhibition [24,25]. In addition, our recent study also found that WSTC could inhibit platelet activation and aggregation in vitro [26]. Further acute randomized controlled trials (RCTs) in Britain have suggested that WSTC attenuated platelet aggregation and activation after a single dose of supplementation [25,27]. However, there has been no randomized controlled trial (RCT) exploring the effects of WSTC on platelet apoptosis. Whether it can inhibit oxidative stress is also unknown, especially in middle-aged and elderly adults.

Therefore, this present randomized placebo-controlled crossover trial aimed to examine the effects of WSTC on oxidative stress and platelet apoptosis in middle-aged and elderly adults.

## 2. Materials and Methods

### 2.1. Subject Recruitment for the Clinical Trial

Volunteers (35–70 years old) were from the health examination center of the First Affiliated Hospital of Sun Yat-sen University and three other community health centers in Guangzhou, Guangdong, China, from March to July 2019. Potential volunteers were interviewed by trained researchers using face-to-face structured screening questionnaires.

The following inclusion criteria were used: (1) men and women from 35 to 70 years old; (2) no serious vascular or hematological diseases; and (3) normal hematuria, liver or kidney function. The following exclusion criteria were used: (1) a history of hypertension, infectious disease, hemostatic disorders, diabetes mellitus or cardiovascular disease (CVD); (2) use of medications known to affect platelets in the past six months; (3) lactating or pregnant women; and (4) allergic to tomatoes or ingredients rich in tomatoes.

### 2.2. Study Design

**Figure 1:**
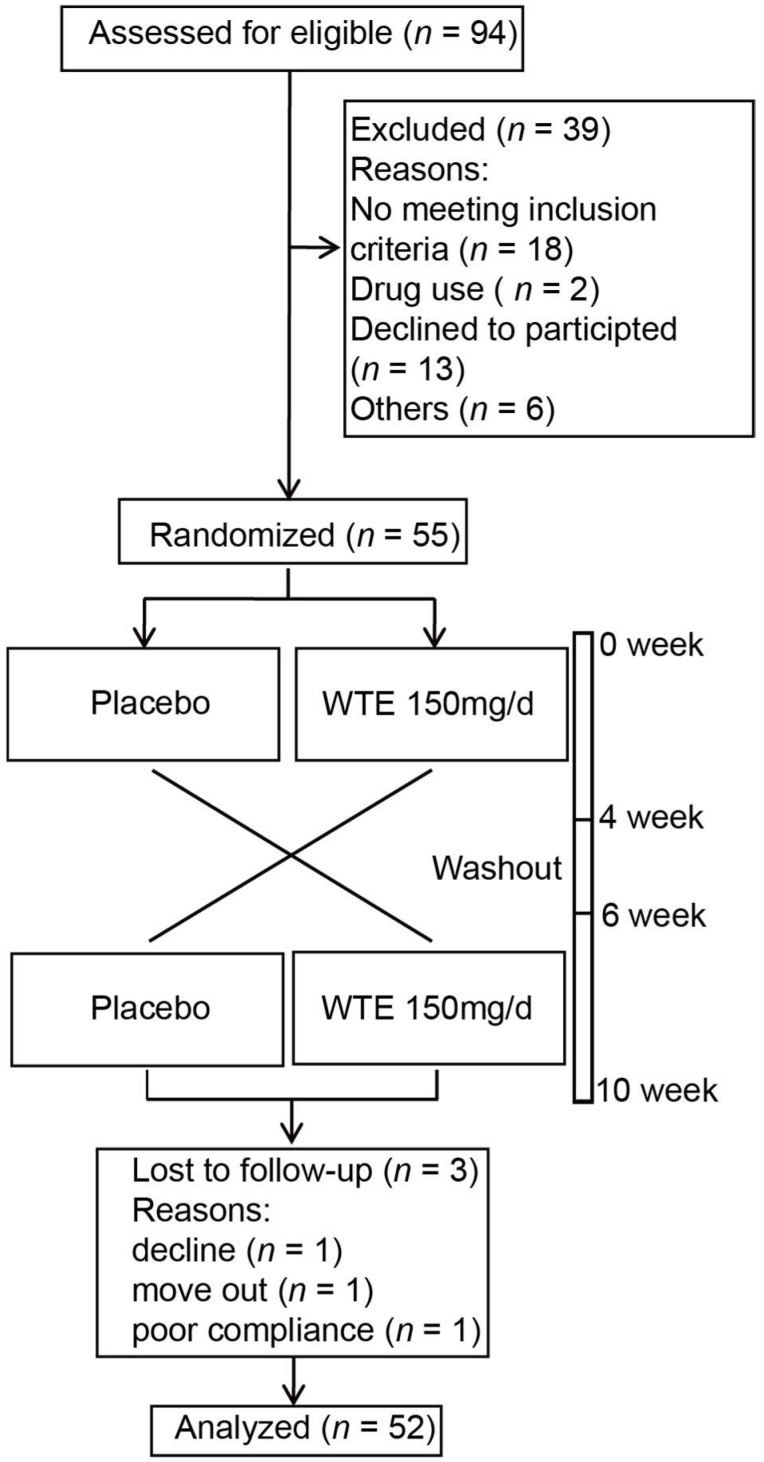
Flow diagram of the subject recruitment and participation procedure.

The study followed a randomized, double-blinded, placebo-controlled crossover design with two 4-week interventions separated by a washout period of 2 weeks (10 weeks in total). In brief summary, the subjects in group 1 took a placebo tablet daily, while the subjects in the group 2 took 150 mg/day of WSTC for 4 weeks. Then, both groups entered a two-week washout period. After that, the two groups exchanged groups. Group 1 took 150 mg/day of WSTC, and group 2 took placebo tablets for 4 weeks (Figure 1).

The volunteers in each gender group were randomly assigned to the two groups via hierarchical randomization. The subjects in both groups took one tablet daily during the two 4-week interventions. All subjects were followed up every two weeks, and the number of returned tablets was recorded to assess the compliance of the subjects. During the trial period, all subjects were instructed to maintain their usual diets and lifestyles but to refrain from the consumption of tomatoes and tomato products. A total of 52 subjects completed the trial (Figure 1). This current trial was conducted in accordance with the Declaration of Helsinki and was approved by the ethics committee of Sun Yat-Sen University (2016 No. 036). All of the subjects gave signed informed consent. This trial is registered at chictr.org as ChiCTR-POR-17012927.

### 2.3. Supplement Preparation

The placebo tablets and WSTC tablets were obtained from BY-HEALTH (Guangdong, China). The WSTC tablets contained 150 mg Fruitflow^®^ Ⅱ developed based on Fruitflow^®^ Ⅰ. Fruitflow^®^ Ⅱ primarily contained adenosine, chlorogenic acid and rutin (Supplemental Table S1) and was approved by the European Food Safety Authority as a cardioprotective functional ingredient [23]. The WSTC and placebo tablets also contained microcrystalline cellulose, lactose, croscarmellose sodium and silica. All WSTC and placebo tablets had the same weight, appearance and packaging. 

### 2.4. Sample Size Planning for the Clinical Trial

Sample size estimation was performed via PASS software (version 15.0, NCSS Inc., Kaysville, UT, USA). A previous study reported that the changes (post-pre) of platelet aggregation after WSTC supplementation were −9.7 ± 4.1% and −3.1 ± 3.9% in the WSTC group and the control group, respectively [27]. Based on a two-tailed α level of 0.05 and β level of 0.10, we performed the two-sample *t*-tests assuming equal variance and determined that 40 subjects should be recruited. Allowing for a 20% dropout rate, at least 50 subjects were required. Since previous studies only explored the effects of WSTC on platelet-related functions, there was no data on the effects of WSTC on oxidative stress for sample size calculations. Based on this, we also performed a post hoc power analysis to extrapolate the sample size according to our current results, as previously described [28]. In summary, our current study found that the MDA levels after WSTC supplementation were 2.83 ± 0.50 nmol/mL and 3.41 ± 0.89 nmol/mL in the intervention group and control group, respectively. According to a two-tailed α level of 0.05 and β level of 0.10, we performed the two-sample *t*-tests assuming equal variance and determined that 34 subjects should be recruited. Allowing for a 20% dropout rate, at least 43 subjects were required. The sample size in our current study was close to the sample size estimation.

### 2.5. Basic Information and Anthropometric Measurement

Basic information and anthropometric measurements were collected as previously described [29]. Basic information was collected via a structured questionnaire by a trained investigator in face-to-face interviews. Height, weight, neck circumference, hip circumference, waist circumference and blood pressure were recorded at weeks 0, 4, 6 and 10. Heart rate and blood pressure (BP) were detected using oscillation monitoring technology (Omron U30 Intellisense, JPN). All measurements were performed under standardized procedures, and the mean value of two measurements was recorded. A 24-h diet record on 3 consecutive days and an international physical activity questionnaire were used to monitor the volunteers’ eating habits and physical activities during the trial. The physical activity data were converted into metabolic equivalents [30], and dietary nutrient intake was calculated according to the Chinese Food Composition Table [31].

### 2.6. Laboratory Measurement

The volunteers fasted for 10–12 h overnight, and venous blood was collected from 8:00–9:00 a.m. on the following day on weeks 0, 4, 6 and 10. The electrical impedance method was used for the routine blood examination. The concentrations of low-density lipoprotein cholesterol (LDL-C), total cholesterol (TC), triglyceride (TG) and creatinine were measured using enzymatic methods. The concentration of serum alanine aminotransferase was determined using the rate method. Serum malonaldehyde (MDA) was determined by the TBA method using commercial kits (catalog no. A003-1-2, Jiancheng, Nanjing, China), and the serum total antioxidant capacity (TAC) was determined by the FRAP method using commercial kits (catalog no. A015-3-1, Jiancheng, Nanjing, China).

The thrombin clotting time (TT), prothrombin time (PT), activated partial thromboplastin time (APTT), and plasma fibrinogen (Fib) estimations were detected by a Sysmex CS-5100 System (Siemens Healthineers, Erlangen, Germany).

### 2.7. Detection of Platelet Aggregation and Activation

As previously described [25,27,32], platelet aggregation was analyzed via the CHRONO-LOG aggregometer (Chrono-log, Havertown, PA, USA). Briefly, 500 μL of fresh platelet-rich plasma (PRP) from citrated blood was incubated at 37 °C for 5 min. Then, PRP (3.0 × 10^8^ platelets/mL) was stimulated by 5 μmol/L ADP or 2 μg/mL collagen on the Chronolog aggregometer at 37 °C with a sample stir speed of 1000 rpm. The maximum reversible platelet aggregation was monitored and recorded.

The expression of P-selectin and PAC-1 on platelets was measured using a CytoFLEX flow cytometer (Beckman Coulter, Brea, CA, USA) as previously described [32,33]. Fresh PRP (5 × 10^6^ platelets/mL) from citrated blood were labeled with FITC-conjugated anti-human CD62P antibody or FITC-conjugated anti-human PAC-1 antibody for 20 min with the stimulation of ADP or collagen. Then, the samples were fixed with 1% paraformaldehyde and analyzed using a CytoFLEX flow cytometer with CytExpert 2.0 (Beckman Coulter, Brea, CA, USA).

### 2.8. Measurement of ROS, ΔΨm and PS Exposure in Human Platelets

Endogenous ROS was measured using DCFH-DA (Sigma-Aldrich, Burlington, MA, USA). Briefly, the platelets were preincubated with DCFH-DA (10 μM) at 37 °C in the dark for 30 min and washed with PIPES. The preincubated platelets in the clinical trial were incubated with or without thrombin (2 unit) for 30 min and detected using flow cytometry. The cells were collected and detected using a Spark^®^ multimode microplate reader (TECAN, Canton of Zurich, Switzerland) at an excitation wavelength of 488 nm and emission wavelength of 525 nm.

Platelet ΔΨm was measured using tetramethylrhodamine methyl ester (TMRM; Abcam, Cambridge, UK). Washed platelets (5 × 10^6^/mL) were preincubated with or without thrombin for 30 min. All of the samples were incubated with TMRM (400 nM) for 20 min at 37 °C in the dark and measured using flow cytometry.

PS exposure was measured using PE-annexin Ⅴ (Becton Dickinson, Franklin Lakes, NJ, USA) according to the manufacturer’s instructions. Prepared samples were incubated with PE-annexin Ⅴ for 15 min at room temperature, and then, PS was measured by flow cytometry within 30 min.

### 2.9. Statistical Analysis

The data were analyzed using SPSS 20.0 statistical software and GraphPad Prism 5.01 software. The data analysis of the individuals in this study followed the intention-to-treat (ITT) principle, as in previous studies [33,34]. All data were presented as the means ± standard errors of the means (SEMs). The data from week 0 and week 6 (after washout) were combined as pretrial (baseline) data, and the data from weeks 4 and 10 were combined as posttrial data in the clinical trial. The comparability of the two groups at baseline was assessed by one-way analysis of variance (ANOVA). ANOVA was also used to determine the significance of the differences between the placebo and WSTC supplementation groups after 4 weeks of intervention. To further determine the effect of WSTC supplementation on oxidative stress and platelet apoptosis, Student’s *t*-tests for paired data were used to examine significant differences before and after WSTC or placebo supplementation in all volunteers, as in previous studies [28,35]. The differences were considered significant at *p* < 0.05.

## 3. Results

### 3.1. Subsection

The mean age of the subjects was 56 years old (44–68 years old), and 30.77% were males. Baseline data were collected before distribution, including sociodemographic data, medical histories, drug use information and anthropometric characteristics (Table 1). There was no significant difference in the anthropometric characteristics, blood lipids or blood glucose between the two groups of volunteers at baseline or during the intervention period (Supplementary Table S2). In addition, there was no significant difference in energy, intake of nutrients or physical activity between the two groups at baseline or after the 4-week intervention (Supplementary Table S3).

### 3.2. Effects of WSTC Supplementation on TAC and MDA in Healthy Middle-Aged and Elderly Adults

**Figure 2:**
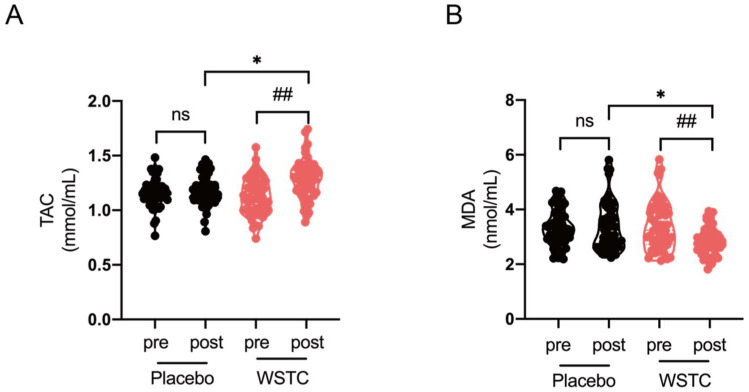
Effects of WSTC supplementation on the serum TAC and MDA levels in healthy middle-aged and elderly adults. (**A**,**B**) Serum TAC and MDA levels. The values are presented as the means ± SEMs. At baseline, there was no significant difference for the TAC and MDA levels between the two groups. * *p* < 0.05, one-way analysis of variance for independent data is used for comparison between the two groups after 4 weeks of intervention. ## *p* < 0.01 vs. baseline in the WSTC group, assessed by a paired Student’s *t*-test. Abbreviations: TAC, total antioxidant capacity, MDA, malonaldehyde, WSTC, water-soluble tomato concentrate and ns, no significance.

There were no significant differences in the serum TAC or MDA levels between the placebo and WSTC groups at baseline. After the 4-week intervention, WSTC supplementation significantly increased the serum TAC levels (*p* < 0.05) and reduced the serum MDA levels (*p* < 0.05) in healthy middle-aged and elderly adults (Figure 2A,B). There were also significant differences in the serum TAC levels (*p* < 0.01) and MDA levels (*p* < 0.01) between the placebo and WSTC groups after intervention. However, there was no significant difference in the serum TAC levels and MDA levels in the placebo group before vs. after the intervention (Figure 2A,B).

### 3.3. WSTC Supplementation Attenuated Platelet ROS Generation in Healthy Middle-Aged and Elderly Adults

**Figure 3:**
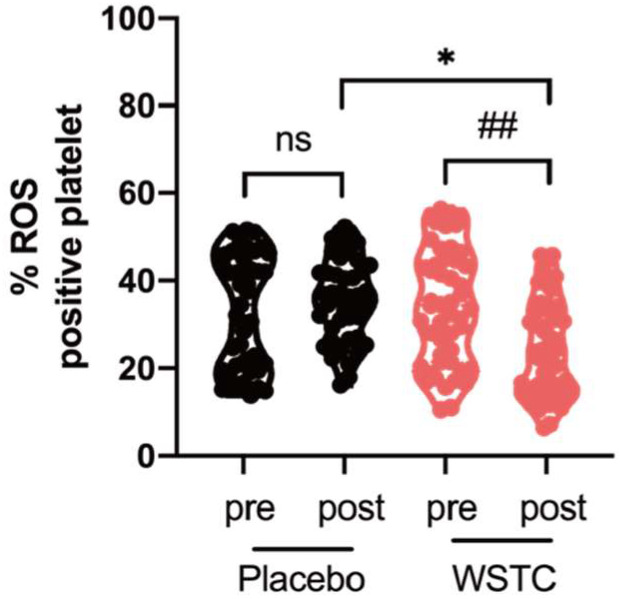
Effect of WSTC supplementation on ROS generation in healthy middle-aged and elderly adults. Human wash platelets were prepared from volunteers before (pre) and after (post) 4 weeks of WSTC or placebo consumption. The washed platelets were pretreated with H2DCF-DA, and ROS was measured by flow cytometry. At baseline, there is no significant difference in the ROS generation between the two groups. * *p* < 0.05, one-way analysis of variance for independent data, is used for comparison between the two groups after 4 weeks of intervention. ## *p* < 0.01 vs. baseline in the WSTC group, assessed by a paired Student’s *t*-test. Abbreviations: ROS, reactive oxygen species, WSTC, water-soluble tomato concentrate and ns, no significance.

As shown in Figure 3, there was no significant difference in circulating platelet endogenous ROS between the two groups at baseline. Circulating platelet endogenous ROS generation in healthy middle-aged and elderly adults was significantly lower in the WSTC supplementation group after 4 weeks of supplementation compared to the placebo group (*p* < 0.05). WSTC supplementation for 4 weeks significantly reduced platelet endogenous ROS generation in healthy middle-aged and elderly adults (*p* < 0.01). In the placebo group, there was no significant change before or after the intervention (*p* > 0.05) (Figure 3).

### 3.4. WSTC Supplementation Attenuated Platelet ΔΨm Dissipation and PS Exposure in Healthy Middle-Aged and Elderly Adults

**Figure 4:**
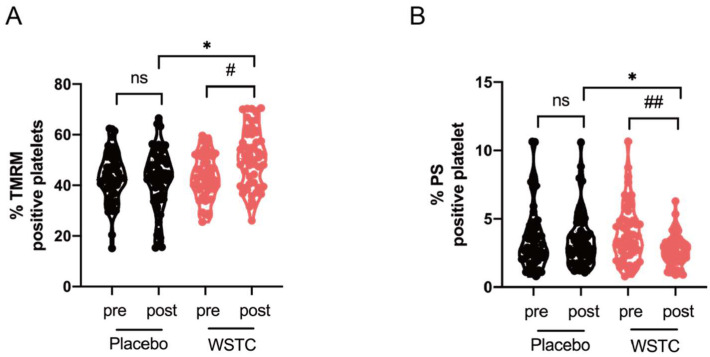
Effect of WSTC supplementation on ΔΨm dissipation and PS exposure in healthy middle-aged and elderly adults. Human wash platelets were prepared from volunteers before (pre) and after (post) 4 weeks of WSTC or placebo consumption. (**A**) Platelet ΔΨm dissipation was measured using TMRM by flow cytometry. (**B**) Annexin V-PE was used to assess platelet PS exposure by flow cytometry. At baseline, there was no significant difference in any variable for ΔΨm dissipation and PS exposure between the two groups. * *p* < 0.05, one-way analysis of variance for independent data was used for comparison between the two groups after 4 weeks of intervention. # *p* < 0.05 and ## *p* < 0.01 vs. baseline in the WSTC group, assessed by a paired Student’s *t*-test. Abbreviations: PS, phosphatidylserine and WSTC, water-soluble tomato concentrate.

At baseline, there were no significant differences in platelet ΔΨm dissipation and PS exposure between the placebo and WSTC groups. Increased circulating platelet ΔΨm has been observed in healthy middle-aged and elderly adults (*p* < 0.05) after 4 weeks of WSTC supplementation (*p* < 0.05) (Figure 4A). Additionally, after WSTC supplementation, WSTC supplementation also markedly attenuated the platelet PS exposure in healthy middle-aged and elderly adults (*p* < 0.05) (Figure 4B). Compared with the placebo group, the platelet ΔΨm in the WSTC group was significantly higher (*p* < 0.05) and platelet PS exposure was significantly lower (*p* < 0.01). However, there was no significant difference in the platelet ΔΨm dissipation and PS exposure in the placebo group before vs. after the intervention (Figure 4A,B).

### 3.5. WSTC Supplementation Attenuated ROS Generation and ΔΨm Dissipation in Thrombin-Treated Platelets

**Figure 5:**
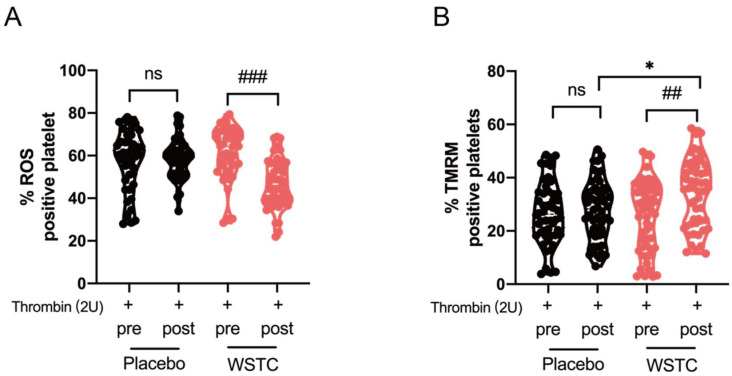
Effects of WSTC supplementation on ΔΨm dissipation and ROS in response to thrombin. Human wash platelets were prepared from volunteers before (pre) and after (post) 4 weeks of WSTC or placebo consumption. (**A**) H2DCF-DA-treated platelets were incubated with thrombin (2 unit), and ROS were measured by flow cytometry. (**B**) The wash platelets were incubated with thrombin (2 unit), and TMRM was used to detect ΔΨm measured by flow cytometry. At baseline, there was no significant difference in any variable about ΔΨm dissipation and ROS between the two groups. * *p* < 0.05, one-way analysis of variance for independent data was used for comparison between the two groups after 4 weeks of intervention. ## *p* < 0.01 and ### *p* < 0.001 vs. baseline in the WSTC group, assessed by a paired Student’s *t*-test. Abbreviations: ROS, reactive oxygen species and WSTC, water-soluble tomato concentrate.

Previous studies have shown that thrombin induced platelet apoptotic events via ROS generation. Therefore, we identified a possible association between ROS and platelet apoptosis using thrombin in the context of WSTC supplementation [36]. At the baseline, the platelet ROS generation and ΔΨm dissipation in the two groups were comparable (*p* > 0.05). WSTC significantly attenuated platelet ROS generation and ΔΨm dissipation in response to thrombin (2 unit) after 4 weeks of supplementation (*p* < 0.05) (Figure 5A,B). Additionally, the platelet ΔΨm in the WSTC group was significantly higher (*p* < 0.01) than that in the placebo group (*p* < 0.01), and platelet ROS generation in the WSTC group was significantly lower than that in the placebo group (*p* < 0.001). There was no significant difference between baseline and the 4-week intervention of the placebo group (*p* > 0.05) (Figure 5A,B).

### 3.6. WSTC Supplementation Inhibited Platelet Aggregation and Activation in Healthy Middle-Aged and Elderly Adults

**Figure 6:**
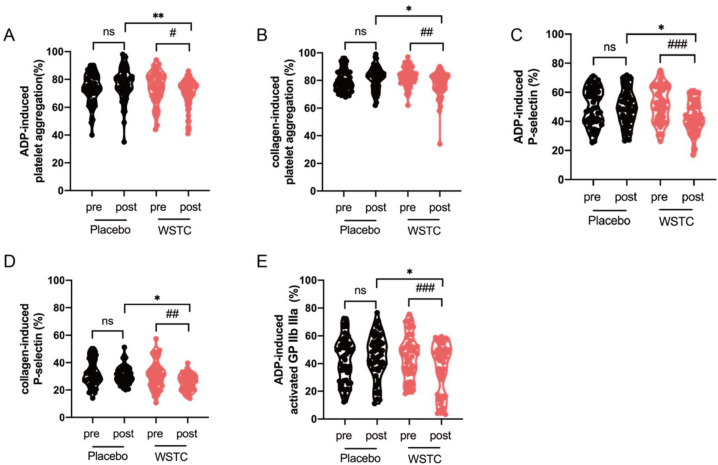
WSTC supplementation inhibited platelet aggregation and activation in healthy middle-aged and elderly adults. Human PRP was prepared from volunteers before (pre) and after (post) 4 weeks of WSTC or placebo consumption. (**A**,**B**) Platelet aggregation was stimulated by ADP or collagen. PRP was stimulated with ADP or collagen. The platelet surface expression of (**C**,**D**) P-selectin and (**E**) PAC-1 was analyzed by flow cytometry. The values are presented as the means ± SEMs. At baseline, there was no significant difference for platelet aggregation and activation between the two groups. * *p* < 0.05 and ** *p* < 0.01, one-way analysis of variance for independent data was used for a comparison between the two groups after 4 weeks of intervention. # *p* < 0.05, ## *p* < 0.01 and ### *p* < 0.001 vs. baseline in the WSTC group, assessed by a paired Student’s *t*-test. Abbreviations: PRP, platelet-rich plasma and WSTC, water-soluble tomato concentrate.

Previous acute studies in Britain have found that WSTC significantly inhibited collagen- and ADP-induced platelet aggregation [25,27]. Therefore, we also measured platelet activation and aggregation stimulated by collagen and ADP in healthy middle-aged and elderly adults. We found that four weeks of WSTC supplementation effectively reduced the platelet aggregation induced by ADP or collagen (*p* < 0.05) (Figure 6A,B). WSTC supplementation in healthy middle-aged and elderly adults markedly attenuated platelet surface P-selectin expression and glycoprotein IIb IIIa activation (PAC-1) induced by ADP or collagen (*p* < 0.05) (Figure 6C–E). There were also significant differences in platelet aggregation induced by ADP or collagen between the placebo and WSTC groups after the intervention (*p* < 0.05) (Figure 6A,B). Compared with the placebo group, platelet surface P-selectin expression and glycoprotein IIb IIIa activation (PAC-1) induced by ADP or collagen in the WSTC group were significantly lower after a 4-week supplementation (*p* < 0.05) (Figure 6C–E).

### 3.7. Safety Evaluation

Previous studies did not observe clinical side effects after 0–7 days of WSTC intervention [27]. We performed 4 weeks of WSTC supplementation for the first time and found that no adverse event was reported during the whole intervention period. In addition, considering the antiplatelet effects of WSTC, we carefully determined whether the clotting pathways were affected by WSTC alongside the antiplatelet effects. We examined the coagulation function of the subjects and found that a 4-week intervention of WSTC did not affect APTT, TT, PT, PT-INR, Fib and PT-R in healthy middle-aged and elderly adults (*p* > 0.05, Table 2). There was no significant difference in the platelet parameters, coagulation function or liver and kidney function in the WSTC group compared to the placebo group (*p* > 0.05, Table 2).

## 4. Discussion

An increasing number of studies now suggest broad protective effects of functional foods against CVD [23,37,38]. The present study used a randomized placebo-controlled clinical trial and showed that 4 weeks of supplementation with WSTC (150 mg/day) increased the antioxidative capacity in serum (e.g., increased TAC and decreased MDA levels) and attenuated circulating platelet ROS generation and apoptosis (e.g., attenuated ΔΨm decrease and PS exposure) in healthy middle-aged and elderly adults. Additionally, the 4 weeks of WSTC supplementation also inhibited platelet activation and aggregation, which was consistent with previous studies [25,27]. Our results suggest that WSTC is a promising agent for ASCVD prevention that exerts its beneficial effects via the control of oxidative stress and platelet apoptosis.

Mitochondrial alterations contribute to the pathogenesis of CVD [39]. Mitochondria also play a central role in platelet metabolism, and mitochondrial membrane depolarization is the initial step of mitochondrial-mediated apoptosis [40]. WSTC supplementation significantly attenuated platelet ΔΨm dissipation in our present study, which suggests that WSTC plays a vital role in reducing early damage to platelet function. Negatively charged PS is exposed to the outer membrane of apoptotic platelets, and it is a central procoagulant factor that accelerates the progression of thrombosis [4,41,42]. PS-positive apoptotic platelets are enhanced in patients with prothrombotic states [43]. Notably, our results also showed that supplementation with WSTC attenuated platelet PS exposure on circulating platelets, which may contribute to thrombotic disease inhibition. In addition, our previous in vitro study support as well that WSTC could modulate platelet ΔΨm dissipation and inhibit Cytochrome c release, caspase activation and PS exposure in H_2_O_2_-treated platelets [44]. These results indicated that WSTC might inhibit mitochondria-dependent platelet apoptosis. These findings are consistent with Xiao et al., who revealed that quercetin, one component of WSTC, inhibited stored platelet apoptosis by increasing the Bcl-2/Bax ratio in a concentration-dependent manner [45]. Notably, nutrients promote apoptosis in cancer cells, but chlorogenic acid, rutin and polyphenols (e.g., resveratrol and curcumin) have to exert an antiapoptotic effect by inhibiting oxidative stress in normal cell lines [46,47]. Whether one or more components of WSTC or only the mixture contribute to protecting platelet apoptosis is worthy of further study. Overall, our data suggest that WSTC supplementation may alleviate the risks of atherothrombosis via attenuating platelet apoptosis in healthy middle-aged and older people.

Oxidative stress is a central pathological mechanism in ASCVD that induces hypertrophic signaling, apoptosis and necrosis [48]. Antioxidant administration reduces oxidative stress-related apoptosis in the pathogenesis of lots of diseases [49,50]. The present study showed that WSTC supplementation effectively increased TAC while decreasing the MDA levels and platelet endogenous ROS generation, which is consistent with other studies showing that the main bioactive components of WSTC possessed antioxidant properties [51,52,53]. Under certain thrombotic conditions, the levels of oxidative stress (e.g., ROS) are enhanced, and platelet redox homeostasis is disrupted; these changes have many important proatherogenic effects, including the stimulation of platelet hyperreactivity and apoptosis [36]. Whether WSTC attenuates oxidative stress-related platelet apoptosis under disease conditions merits further investigation. Our present study shows that WSTC may exert its beneficial effects on ASCVD prevention via the control of oxidative stress in healthy middle-aged and elderly adults. 

Superoxide anion and H_2_O_2_ are the main ROS. The superoxide anion may be converted into H_2_O_2_ to modulate intraplatelet redox physiological signaling and stimulate platelet apoptosis [12,54]. Previous studies revealed that H_2_O_2_-induced ROS production was consistent with the changes observed in apoptosis [49]. Our previous in vitro study showed that pretreatment with WSTC dose-dependently attenuated ROS generation in H_2_O_2_-treated platelets, and a combined treatment with WSTC and NAC did not result in significant differences in the apoptosis levels compared with the WSTC treatment alone [44]. A further in vitro study showed that WSTC could increase the expression level of LC3Ⅱ/Ⅰ in H_2_O_2_-treated platelets. Furthermore, the effect of WSTC on decreasing ΔΨm depolarization in H_2_O_2_-treated platelets was reversed by an autophagy inhibiter (3-MA) [44]. These indicated that WSTC can significantly reduce the H_2_O_2_-induced platelet oxidative damage by promoting autophagy in vitro. Therefore, oxidative stress could potentially be essential in inhibiting platelet apoptosis by WSTC. The attenuation of ROS-scavenging activity only partially reflects the antioxidant potential, and the antioxidant properties of WSTC may also contribute to its antiapoptotic benefits, which are worthy of further investigation. 

WSTC, authorized by the European Food Safety Agency, is approved to take 150 mg/day in the format of powder, tablet or capsule [23]. Therefore, the dose of 150 mg/day was used in this clinical trial. WSTC was first found to have antiplatelet functions (e.g., anti-activation and anti-aggregation effects) in Britain. Previous acute clinical trials found that WSTC supplementation could attenuate platelet activation and aggregation after 3 h or 7 h [27,55]. To further verify the accuracy of this result, we also explored the effect of WSTC on platelet function in this study. Notably, our study found that 150 mg/day of WSTC has remarkably inhibited platelet activation and aggregation induced by collagen or ADP after a 4-week intervention in healthy middle-aged and elderly adults. These are consistent with previous studies in Britain. Taking antiplatelet drugs such as aspirin and warfarin can reduce the risk of cardiovascular events, but it may also increase the risk of bleeding [56]. Due to the antiplatelet effects of WSTC, we also explored whether WSTC could affect the coagulation function. In our clinical trial, WSTC does not affect the coagulation function related to the prothrombin system after 4 weeks of WSTC supplementation. Our previous animal experiment also found that WSTC could not prolong the bleeding time of mice [26]. Interestingly, our previous study found that the inhibitory effect of 4 weeks of WSTC on platelet aggregation and activation can be eliminated after a 2-week washout period [57]. In addition, we also found that a 4-week intervention of WSTC had no effect on the liver function or kidney function in healthy middle-aged and elderly adults, and this was consistent with previous studies [23,58]. Although whether there is a risk of bleeding after long-term use still needs to be confirmed by further studies with larger sample sizes and longer intervention durations, the current results suggest that WSTC may be used as a potentially safe and reliable nutrient supplement for regulating platelet function.

### Strengths and Limitations

There are some strengths and certain limitations in this study. The major strength of this study is the design of a double-blinded, randomized, placebo-controlled, crossover trial. Another strength is that we tested the platelet ROS generation, PS exposure and ΔΨm at baseline and during follow-up, which should be completed in two hours after blood collection. For the first time, we explored the effect of WSTC on oxidative stress and platelet apoptosis in healthy middle-aged and elderly adults. This could be another advantage. As in previous studies [28,34], we also maintain all usual dietary intake and physical activities via questionnaires to control the effects of a confounding bias. Nevertheless, there are still some limitations present in this study. One of the limitations is that, due to the complex composition of WSTC, it is unlikely for us to measure the serum levels of WSTC or its metabolites. The results of this study only indicated a potential benefit of WSTC on the early prevention of CVDs in healthy middle-aged and elderly adults, and the prevention of WSTC in individuals with metabolic diseases merits further clinical study. This may be another limitation of our study.

## 5. Conclusions

The results showed that four weeks of WSTC supplementation could attenuate oxidative stress and platelet apoptosis in healthy middle-aged and elderly adults. In addition, our study further added evidence that WSTC could safely and effectively reduce platelet aggregation and activation in healthy middle-aged and elderly adults. Therefore, our current results indicate that the beneficial effects of WSTC may have a potential role in the early prevention of ASCVD.

## Figures and Tables

**Table 1:** Baseline characteristics.

	N = 52
Age, (years)	56.13 ± 1.01 ^a^
Gender (male/female)	16/36
**Education attainment**	
Primary school	2(3.85%)
Middle school	23(44.23%)
College	27(51.92%)
**Occupations**	
Sales/workers/farmers	18(34.61%)
Professionals/technicians	29(55.76%)
Others	5(9.62%)
**Anthropometrics**	
Weight (kg)	64.17 ± 1.70
BMI (kg/m^2^)	24.57 ± 0.46
NC (cm)	34.40 ± 1.08
WC (cm)	84.99 ± 1.59
WHR	0.88 ± 0.01
SBP (mmHg)	118.32 ± 2.19
DBP (mmHg)	77.19 ± 1.47
**Lifestyle factors**	
Current smoking	2(3.8%)
Regular alcohol drinking	11(21%)

^a^ The results are presented as the mean ± standard error of the mean for continuous variables and n (%) for categorical variables. Abbreviation: BMI, body mass index, WC, waist circumference, NC, neck circumference, WHR, waist-to-hip ratio, DBP, diastolic blood pressure and SBP, systolic blood pressure.

**Table 2:** Blood chemistry and plasma clotting times at baseline and after the 4-week treatment ^b^.

	Placebo (*n* = 52)		150mg WSTC (*n* = 52)	
	Baseline	4 Weeks	Baseline	4 Weeks
**Liver function**				
ALT (U/L)	21.08 ± 1.57 ^a^	21.27 ± 2.03	21.75 ± 1.97	20.55 ± 1.51
Total protein (g/L)	74.19 ± 0.50	73.37 ± 0.45	73.58 ± 0.56	73.16 ± 0.53
Albumin (g/L)	47.05 ± 0.32	45.97 ± 0.33	46.63 ± 0.36	45.55 ± 0.35
Albumin/Globulin	1.77 ± 0.04	1.69 ± 0.03	1.76 ± 0.03	1.68 ± 0.03
Renal function				
Urea (mmol/L)	4.86 ± 1.42	4.97 ± 0.15	5.01 ± 0.17	4.82 ± 0.14
Creatinine (μmol/L)	78.35 ± 2.44	78.29 ± 2.30	79.21 ± 2.36	78.44 ± 2.38
**Plasma clotting times**				
Prothrombin time (s)	10.81 ± 0.73	11.26 ± 0.81	10.90 ± 0.71	11.16 ± 0.73
APTT (s)	28.75 ± 0.24	28.64 ± 0.28	28.68 ± 0.28	28.48 ± 0.25
Thrombin time (s)	18.76 ± 0.09	18.76 ± 0.13	18.77 ± 0.10	18.91 ± 0.11
Fibrinogen (g/L)	2.98 ± 0.06	3.03 ± 0.08	3.01 ± 0.07	2.92 ± 0.08
**Platelet parameters**				
PLT (10^9^/L)	244.20 ± 9.05	253.53 ± 9.90	244.12 ± 8.71	250.69 ± 9.27
MPV (fl)	10.20 ± 0.15	10.18 ± 0.13	10.17 ± 0.15	10.22 ± 0.15

^a^ Mean ± SEM (all such values). ^b^ A one-way analysis of variance for independent data was used for comparison between the two groups at baseline and after 4 weeks of intervention. There was no significant difference for any variable concerning the blood chemistry and plasma clotting times between the two groups at baseline and after the 4-week intervention. Abbreviations: ALT, alanine aminotransferase, PT-R, prothrombin time ratio, PLT, plaque level test, MPV, medial plaque volume and APTT, activated partial thromboplastin time.

## Data Availability

Data, protocol of this study, plan of statistical analysis and informed consent will be made available upon reasonable request via email to the corresponding author.

## Supplementary Materials

The following supporting information can be downloaded at https://www.mdpi.com/article/10.3390/nu14163374/s1: Table S1: The composition of water-soluble tomato extract, Table S2: Anthropometric measurements, lipid profiles and glucose at baseline and 4 weeks after treatment, Table S3: Daily dietary intakes and physical activity at baseline and 4 weeks after treatment.
