# Supplementary material for: Water-Soluble Tomato Concentrate, a Potential Antioxidant Supplement, Can Attenuate Platelet Apoptosis and Oxidative Stress in Healthy Middle-Aged and Elderly Adults: A Randomized, Double-Blinded, Crossover Clinical Trial
Source: Nutrients. 2022 Aug 17;14(16):3374. doi: 10.3390/nu14163374 (PMC9412583; doi:10.3390/nu14163374)
Supplement: Supplementary file 1 [file nutrients-14-03374-s001.zip › nutrients-1826223-supplementary.pdf]

Supplemental Table S1 The composition of water-soluble tomato extract

**Components**	**Content**
**Physicochemical parameters**	
Moisture content (%)	3.3
Ash content (%)	1.9
**Microbe Index**	
Total number of colonies (CFU/g)	≤1000
Mold and yeast (CFU/g)	≤100
Escherichia coli	-
Salmonella	-
Staphylococcus aureus	-
Pseudomonas aeruginosa	-
**Active ingredient**	
nucleosides, polyphenols and flavonoids (mg/g)	23

Supplemental Table S2 Anthropometric measurements, lipid profiles and glucose at baseline and 4 weeks after treatment [b]

	Placebo (n=52)		150mg WSTC(n=52)	
	Baseline	4 weeks	Baseline	4 weeks
Weight (kg)	63.85±1.68[a]	63.40±1.66	64.21±1.70	63.61±1.71
BMI (kg/m^2)	24.45±0.45	24.29±0.45	24.59±0.46	24.33±0.46
NC (cm)	35.24±1.27	33.35±0.44	36.26±1.60	34.47±1.06
WC (cm)	83.31±1.88	84.92±1.13	82.81±1.78	84.09±1.61
HC (cm)	95.13±0.90	94.85±0.93	95.13±0.89	95.49±0.91
WHR	0.87±0.02	0.89±0.01	0.87±0.02	0.88±0.01
TG (mmol/L)	1.53±0.09	1.56±0.15	1.47±0.09	1.48±0.09
TC (mmol /L)	5.07±0.12	5.11±0.12	5.02±0.11	4.97±0.13
HDL-c (mmol /L)	1.34±0.04	1.29±0.04	1.32±0.04	1.30±0.04
LDL-c (mmol /L)	3.38±0.11	3.43±0.11	3.35±0.10	3.32±0.12
FBG (mmol/L)	5.15±0.07	5.23±0.09	5.16±0.08	5.19±0.08

[a] Mean ± SEM (all such values).

[b] A one-way analysis of variance for independent data are used for comparison between the two groups at baseline and after 4 weeks of intervention. There are no significant differences for anthropometric measurements, lipid profiles and glucose between the two groups at baseline and after 4 weeks of intervention.

Abbreviation: BMI body mass index, NC neck circumference, WC waist circumference, HC hip circumference, WHR waist-to-hip ratio, TC total cholesterol, HDL-c high density lipoprotein cholesterol, LDL-c low density lipoprotein cholesterol, TG total triglyceride, FBG fasting blood glucose.

Supplemental Table S3 Daily dietary intakes and physical activity at baseline and 4 weeks after treatment [b]

	Placebo (n=52)		150mg WSTC(n=52)	
	Baseline	4 weeks	Baseline	4 weeks
Total energy(kcal/d)	1620.96±104.48 [a]	1533.05±70.59	1590.57±86.16	1486.48±70.59
Total protein(g/d)	72.80±4.19	68.98±3.08	73.02±4.32	67.13±3.34
Carbohydrates (g/d)	207.90±13.31	210.47±13.59	214.81±13.08	200.22±12.21
Total lipids (g/d)	54.84±5.50	45.89±2.84	49.25±3.86	46.39±2.81
Cholesterol (mg/d)	424.68±36.07	398.15±22.47	457.68±36.07	385.63±30.54
Dietary fiber (g/d)	12.45±1.11	11.70±0.84	11.29±0.91	10.99±0.86
Vitamin C (mg/d)	113.10±9.87	128.69±11.10	123.35±12.82	121.67±10.63
Vitamin A (ug retinol equivalent/d)	661.38±59.34	625.63±63.90	869.99±161.48	663.30±57.02
Vitamin E (mg/d)	14.56±1.41	13.72±1.02	14.21±1.42	12.71±1.13
Physical activities (MET-min/week)	6883.57+561.48	7086.64±624.20	7101.01±544.80	7216.35±719.31

[a] Mean ± SEM (all such values).

[b] A one-way analysis of variance for independent data are used for comparison between the two groups at baseline and after 4 weeks of intervention. There are no significant differences for daily dietary intakes and physical activity between the two groups at baseline and after 4 weeks of intervention.

Abbreviation: SEM, standard error of mean, MET metabolic equivalent.
